# Multi-Epitope-Based Vaccine Candidate for Monkeypox: An In Silico Approach

**DOI:** 10.3390/vaccines10091564

**Published:** 2022-09-19

**Authors:** Sayed Aliul Hasan Abdi, Amena Ali, Shabihul Fatma Sayed, Abutahir, Abuzer Ali, Prawez Alam

**Affiliations:** 1 Department of Pharmacy, Al Baha University, Al Baha 1988, Saudi Arabia; 2 Department of Pharmaceutical Chemistry, College of Pharmacy, Taif University, P.O. Box 11099, Taif 21944, Saudi Arabia; 3 College of Nursing, University College Farasan Province, Jazan University, Jazan 54943, Saudi Arabia; 4 Hakikullah Chaudhary College of Pharmacy, Gharighat, Gonda 27132, India; 5 Department of Pharmacognosy, College of Pharmacy, Taif University, P.O. Box 11099, Taif 21944, Saudi Arabia; 6 Department of Pharmacognosy, College of Pharmacy, Prince Sattam bin Abdulaziz University, P.O. Box 173, Al Kharj 11942, Saudi Arabia

**Keywords:** monkeypox, vaccine, immunoinformatics, multi-epitope

## Abstract

Currently, there are limited treatment options available for the monkeypox disease. We used a computational strategy to design a specific antigenic vaccine against pathogens. After using various immunoinformatic tools and filters, cytotoxic T-cell lymphocyte (CTL)-, helper T-cell lymphocyte (HTL)-, and interferon gamma (IFN-γ)-inducing epitopes, which comprised the vaccine, in addition to other parameters, such as antigenic and allergic profiles, were assessed to confirm the safety of the vaccine. However, vaccine interaction and stability with Toll-like receptors (TLRs) were assessed by dynamic simulation methods, and it was found that the constructed vaccine was stable. In addition, C-IMMSIM tools were used to determine the immune-response-triggering capabilities of the vaccine. These immunoinformatic findings reveal that constructed vaccine candidates may be capable of triggering an efficient immune response for monkeypox viral infections. However, experimental evaluation is required to verify the safety and immunogenic profile of constructed vaccines.

## 1. Introduction

Monkeypox is a viral zoonotic disease. Although it is clinically less severe than smallpox [1]. As per the WHO’s report, the fatality ratio of monkeypox is around 3–6% [2]. This disease of is global public health importance because it may affect the entire world, like coronavirus [3]. In May 2022, several cases were reported in various countries, and studies are underway to better understand the transmission patterns, source of infection, and epidemiology [2]. Monkeypox genome is ~197 kb and has ~190 open reading frames (ORFs). In addition, it has been reported that the monkeypox genome may be responsible for immunomodulation [4,5]. Monkeypox virus is a double-strand DNA virus surrounded by a lipoprotein envelope, approximately 200–500nmin size, and comes under the family of poxviridae [6]. The frequency and geographic distribution of the monkeypox virus have been reported across west and central Africa in recent years. In most cases, infected individuals are <40 years of age [7,8]. The transmission of the monkeypox virus can occur through aerosols. However, direct or indirect exposure to live or dead animals may be a risk factor in the transmission of monkeypox disease [6]. In addition, poverty and courtier’s civil unrest compel people to hunt small animals for consumption, and these animals may carry the monkeypox virus [9]. In the current scenario, monkeypox cases have been reported in various parts of the world, such as India, UAE, Saudi Arabia and the United States. However, there are various types of monkeypox strains that have been reported.

According to the WHO, smallpox vaccine could be considered for the prevention and treatment of monkeypox. However, a specific vaccine for monkeypox is still under investigation. If the vaccine is designed as per the conventional method, large proteins are generally used, and it may cause mild to severe allergic reactions because of the presence of an inappropriate antigen [10,11]. Although, a peptide-based multi-epitope vaccination with small antigenic peptide fragments known as epitopes may be able to bypass these restrictions. The epitope, which is the antigenic portion of the virus or any other pathogen, may be identified by innate immunity and the host immune system may elicit an immune response against it. Additionally, Toll-like receptors have an important role in it [12]. In this study, we used an immunoinformatic approach to design a vaccine for the target FASTA protein sequence. The final vaccine was made up of helper T-cell lymphocytes (HTL), cytotoxic T-cell lymphocytes (CTL), and interferon (IFN) gamma. In addition, the physicochemical properties, molecular docking, and thermodynamic stability of the constructed vaccine were also assessed for the safety and efficacy of the vaccine.

## 2. Materials and Methods

### 2.1. Monkeypox Protein Sequence and the Analysis of the Phylogenetic Tree

Various monkeypox strains were screened in the NCBI database, (https://www.ncbi.nlm.nih.gov/ (last accessed on 14 August 2022) and the FASTA format was used for storage. Using the Clustal Omega tool server, (https://www.ebi.ac.uk/Tools/msa/clustalo/, last accessed on 14 August 2022) sequences were aligned and a phylogenetic tree was created [12].

### 2.2. Physiochemical and Antigenic Properties of the Target Protein

For the identification of most antigenic proteins, the protein sequence of a selected strain was submitted to VaxiJen v2.0 Server (http://vaxijen/VaxiJen/VaxiJen.html/, last accessed on 14 August 2022) with the default server. The physicochemical properties of the vaccine were assessed using the ExPASy ProtParam tool server, (https://web.expasy.org/protparam//, last accessed on 14 August 2022) [13,14].

### 2.3. Prediction and Assessment of T-Cell Epitopes

For the prediction of T cell epitopes, the FASTA sequence of a selected strain was submitted to the NetCTL-1.2 server, (https://services.healthtech.dtu.dk/NetCTL-1.2/, last accessed on 14 August 2022) by identifying HLA 12 CLASS I supertypes, and the further IEDB SMM method was used to assess the binding affinities of the epitopes with MHC class I alleles. The epitope was selected on the basis of its ranking. For NetCTL-1.2, server thresholds were set at 0.75. The TAP transport efficiency and the proteasomal C-terminal cleavage were set at 0.05 and 0.15, respectively [15,16,17].

### 2.4. Prediction of HTL Epitope

The Net MHCII pan 3.287 server (https://services.healthtech.dtu.NetMHCIIpan-3.2, last accessed on 14 August 2022) was applied for the analysis of human leukocyte antigen class II DRB1 alleles 01:01, 03:01, 04:01, 07:01, 08:01, 08:03, 10:01, 11:02, 12:01, 13:02, 14:01, and 15:01. The server Net MHCII pan 3.287 is based on artificial neuron networks, which predict peptides that may bind to HLA-DQ, HLA-DR, and HLA-DP alleles. The prediction of the epitope is based on the affinity of the receptor. The server gives a percentile rating for each expected output based on the affinity of the receptor. On the basis of percentile ratings ≤2%, ≤2–10%, and ≥10%, epitopes were categorized by strong, weak, and non-binder. The antigenicity of each epitope was evaluated by VaxiJen v.2.0 server, and allergies were predicted by AllerTOP v.2.088 (https://AllerTOP///, last accessed on 14 August 2022) [13,16,18,19].

### 2.5. Prediction of Interferon-Gamma-Inducing Epitopes and Population Coverage

The natural killer cells are activated by cytokines and macrophages to produce immunity against viral and bacterial infections. To predict the IFN-epitope, the IFN-epitope server (http://crdd.osdd.net/ragha-va/ifnep-itope/, accessed on 14 August 2022) was used. The prediction was assessed using a motif hybrid technique and a support vector machine (SVM) on the basis of more than 10,000 validated helper T-cell epitopes. Because of regional differences in HLA allele distribution, the IEDB population coverage (http://tools.iedb.org/population, accessed on 14 August 2022) was used for this global assessment of HLA alleles [20,21].

### 2.6. Construction of the Vaccine, Structure Modelling, and Validation

The AYY and GPGPG linkers were used for selected CTL, HTL, and IFN epitopes. In addition, CTB was added as an adjuvant via the EAAAK linker to the N-terminal. For the assessment of secondary features of the vaccine, the SOPMA server (https://npsa-prabi.ibcp.fr, last accessed on 14 August 2022) predicted strands, alpha-helical regions, beta turns, and random coils. In addition, for the creation of three-dimensional models, the trRosetta (transform-restrained Restta) online tool (https://yanglab.nankai.edu.cn/trRosetta/, accessed on 14 August 2022) was used, and to refine the model, the Galaxy Refine web server was used. Furthermore, the structural validity of the constructed vaccine was evaluated on the basis of the ERRAT program, a Z-score, and the Ramachandran plot by using the PROCHECK server, (https://www.ebi.ac.uk/thornton-srv/PROCHECK/, last accessed on 14 August 2022) [22,23,24,25].

### 2.7. Physicochemical and Antigenic Assessment of the Constructed Vaccine

The VaxiJen v2.0 web tool was used to assess the antigenicity of the constructed vaccine. For allergy prediction, AllergenFP v.1.089 (https://pharmfac.net/AllergenFP/ (last accessed on 14 August 2022) and AllerTOP v.2.088 servers were used. For the assessment of physiochemical properties of the constructed vaccine, the ExPASy ProtParam tool server, (https://protparam/, last accessed on 14 August 2022) was used. The SignalP 4.1 servers, (https://services.healthtech.dtu.dk/service.SignalP-4.1Lyngby, last accessed on 14 August 2022) were used to check signal peptides [13,14]

### 2.8. Prediction of B-Cell Epitopes

The ElliPro server of IDEB, http://tools.iedb.org/ellipro/ (last accessed on 14 August 2022) was used to predict linear and conformational B-cell epitopes. The ElliPro server has three algorithms that calculated the protein’s ellipsoid shape, calculated the protrusion index (PI) of the residues, and clustered nearby residues according to PI values [20,21].

### 2.9. Molecular Docking of a Refined Model

To calculate the binding affinity and interaction patterns between the constructed multi-epitope vaccine and Toll-like receptor 2 and 4 (PDB: 2Z80 and 2Z62, respectively), the structures were retrieved from the RCSB PDB database in PDB format. For molecular docking, the PatchDock server was used, and the FireDock server (http://FireDock, last accessed on 14 August 2022) was used for the refinement of the best docked complex. For the protein–protein molecular docking, the PatchDock server (https://PatchDock, last accessed on 14 August 2022) calculated the surface fix coordinating scores, separating scores, and the portrayal of atomic shapes. This algorithm computed the TLRs and the vaccine molecules into small patches in agreement with the surface. These small patches resembled distinctive shapes, that can visually separate puzzle pieces. The most effective vaccine–TLRs’ complex structure was selected based on the lowest docking energy score [26,27]. Furthermore, molecular interactions were visualized through Discovery Studio 2022.

### 2.10. In Silico Immune Simulation

Immune stimulation, immunogenicity testing, and the determination of the immune response profile for a constructed vaccine were conducted using the C-IMMSIM website. The C-IMMSIM server uses machine-learning techniques to predict immune responses based on three compartments: lymph nodes, thymus, and bone marrow. The entire simulation was run for 800 runs [28,29].

### 2.11. Molecular Dynamics Simulation

The Gromacs software, Uppsala university, Sweden was used to conduct molecular dynamics simulations of the vaccine/TLR complexes. Additionally, an OPLS-AA/L all-atom force field was used, and a topology file was generated. Each TLR complex was cleaned and optimized for hydrogen bonding, according to protocol. The system was neutralized by the addition of Na salt, and the temperature was kept at 310 K. Before the beginning of dynamics simulations, energy minimization was performed to confirm that the system had proper geometry and that there was no steric clash. There were two phases during energy minimization. The first phase was NVT (constant number of particles, volume and temperature), and the second phase was NPT (constant number of particles, pressure and temperature). Finally, the molecular dynamics simulation ran for a period of 10 ns. The trajectories were analyzed for RMSF, RMSD, Rg, and hydrogen bond values, to reveal the stabilities of the structured vaccine complexes with TLR2 and TLR4 [30,31].

## 3. Results

### 3.1. Analysis ofPhylogenetic Tree

**Figure 1:**
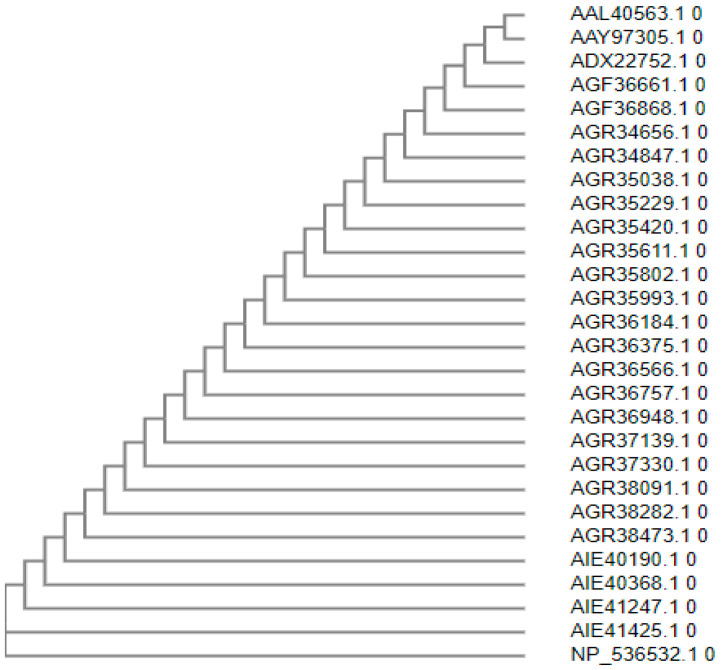
Phylogenetic tree of monkeypox.

The protein sequences of monkeypox from different countries were related to one another. As a result, the monkeypox vaccine may be effective against all strains shown in Figure 1.

### 3.2. Physiochemical and Antigenic Properties of Target Protein

The predicted VaxiJen v2.0 antigen score for a selected monkeypox strain was 0.531, which signifies that the sequence potentially had an antigenic property. The physiochemical properties of the selected monkeypox strain indicate that the selected strain had an instability index score of 44.95, with an aliphatic index and a negative grand average of hydropathicity (GRAVY) value of 87.89 and −0.367, respectively (Supplementary Table S1). The target monkeypox strain had 304 amino acids with a molecular weight of 35,278.02 kDa. The estimated half-life was predicted to be 30 h for mammalian reticulocytes, >20 h for yeast, and >10 h for *Escherichia coli*.

### 3.3. Prediction and Assessment of T-Cell Epitope

The CTL epitope may remove infected virus cells, induce cellular immunity, and decrease circulating viruses. However, HTL epitopes have the capacity to induce both humoral and cellular immunological responses, as well as to stimulate the production of antibodies by B-cells. Therefore, an effective vaccine should have helper T-cell lymphocyte epitopes and receptor-specific cytotoxic T-cell lymphocyte epitopes. Furthermore, the Infectious Disease Epidemiology Bureau’s (IDEB) stabilized matrix method (SMM) was used for CTL epitope prediction, and HTL epitope prediction was conducted by the Net MHCII pan 3.2 server (Supplementary Tables S2 and S3). The predicted epitope sequences were screened for antigenicity, non-allergenicity, high-binding affinity, and for MHC (MHC-I and MHC-II) alleles. The details are given in Supplementary Table S4.

### 3.4. Prediction of Interferon-Gamma-Inducing Epitopes and Population Coverage

The predicted IFN-γ epitope is given in Supplementary Table S5. According to the IDEB Population Coverage tool the epitope chosen for our investigation may cover 100% of the global population.

### 3.5. Construction of Vaccine, Structure Modelling, and Validation

**Figure 2:**
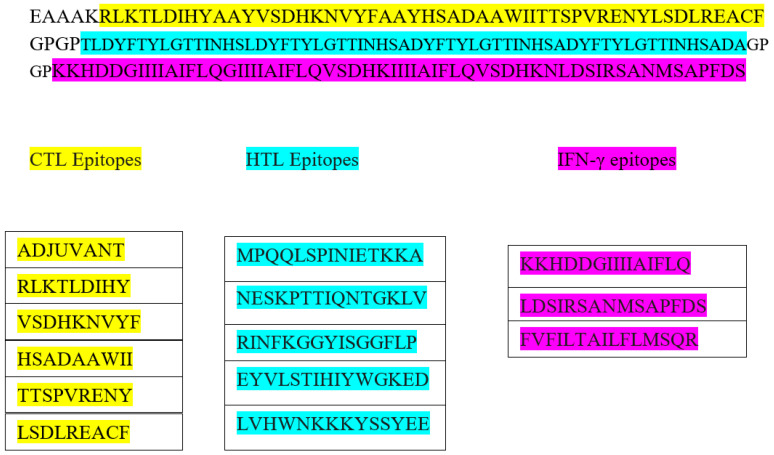
The schematic presentation of constructed vaccine with CTL- in yellow, HTL-in green, and IFN-inducing epitopes in purple, and adjuvant and linker are shown in white color.

**Figure 3:**
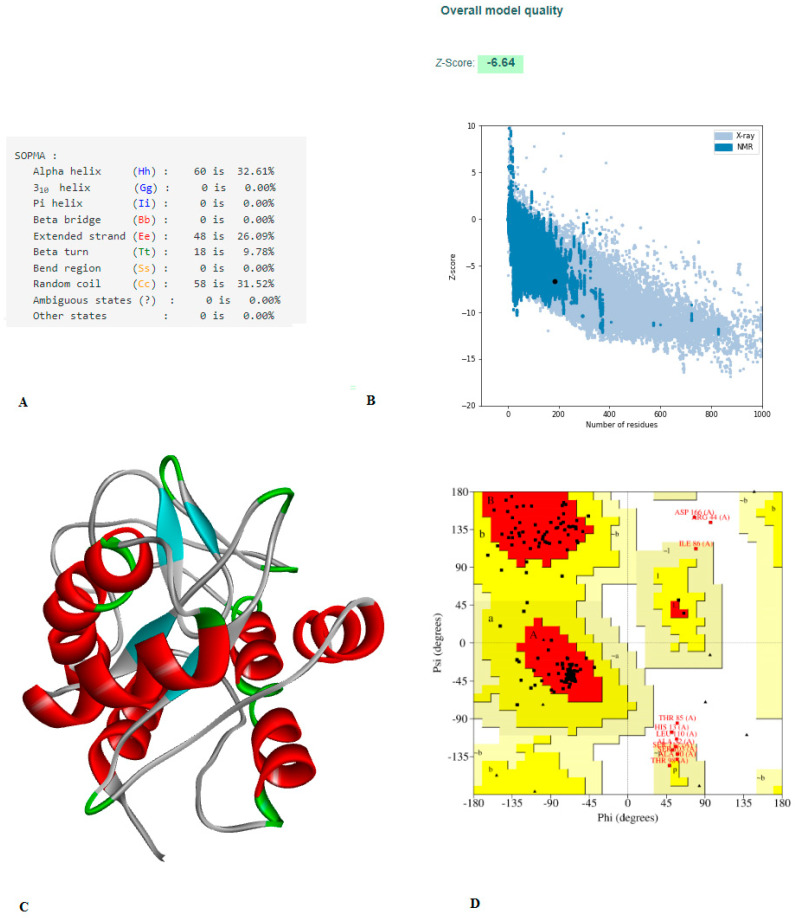
(**A**) The vaccine’s secondary structural characteristics. (**B**) 3D structure validation of with Z-scores is −6.64, evaluated by Pro-SA server. (**C**) 3D model of structured vaccine. (**D**) Using the PROCHECK server, an analysis of the Ramachandran plot revealed that 80.7%, 12.7%, 3.0%, and 3.6% of the residues were found in the favored, additionally allowed, allowed, and disallowed, respectively.

The final vaccine design is depicted schematically in Figure 2. The constructed vaccine had a length of 207 amino acids, and the secondary structure prediction included 23.19% alpha-helical region, 27.54% extended strain, 8.70% beta turns, and 40.58% random coil (details of SOPMA result are shown in Supplementary Figure S1). The five finalized 3D model of the vaccine was constructed by the trRosetta web server. The highest TM-score (0.22, Supplementary Figure S2) was selected for refinement on the Galaxy Refine web server. A TM-score of 0 to 1, or greater than 0.5, indicated that the model had been corrected. The highest TM-score model was assigned to the Galaxy Refine web server, and the refined model was used for the analysis of the Z-score and Ramachandran plot on the SAVESv6.0 server (https://saves.mbi.ucla.edu/, accessed on 14 August 2022). The refined model on the Galaxy Refine web server was selected on the basis of different parameters, such as global distance test-high accuracy GDT-HA (0.968), RMSD (0.35), and MolProbity (2.19). The details of each refined model of the Galaxy Refine web server are given in Supplementary Table S6. The clash score and poor rotamer score for the predicted model were 11.8 and 0.0, respectively. For the assessment of the Z-score of a selected refined model, the Pro-SA web server was used. The results of the Pro-SA web server were found to be within an acceptable range, which demonstrated the reliability of the model. The Ramachandran plot analysis revealed that 80.7%, 12.7%, 3.0%, and 3.6% of residues were in regions that were favored, additionally allowed, allowed, and disallowed (Figure 3D and Supplementary Table S7). The ERRAT value of the refined models was found to be 81.39 (Supplementary Figure S3). A protein model with an ERRAT score greater than 50 was considered to be of excellent quality.

### 3.6. Physicochemical and Antigenic Assessment of the Constructed Vaccine

The predicted score for the constructed vaccine by the VaxiJen v2.0 server was 0.685, which confirms that the candidate vaccine is antigenic. In addition, allergenicity was assessed by the AllerTOP v.2.0 and the AllergenFP v.1.0 server which was found to be non-allergenic (Supplementary Figure S4). For the assessment of physiological properties of the structured vaccines, the ExPASy server was used. The theoretical Pi value and aliphatic index were found to be 5.71 and 97.17, respectively, which indicates that a structured vaccine candidate is thermostable. In addition, the estimated half-life was found to be 1 h for mammalian reticulocytes, 30 min in yeast, and >10 h in *E. coli*. The hydropathicity (GRAVY) score was 0.073, which may suggest that the candidate is hydrophilic in nature and may have the capacity to interact with the aqueous environment (Supplementary Table S8). The instability index was estimated at 35.65, reflecting the stable nature of the protein. As per assessment with SignalP 4.1, the constructed vaccine does not contain any type of single peptide (Supplementary Figure S5).

### 3.7. Prediction of B-Cell Epitopes

Through the release of antibodies, B-cells play a crucial part in humoral immunity. Long-lasting immunity can be achieved by the B-cell receptor recognizing a B-cell epitope. The epitope server has ten linear and four conformational epitopes (Supplementary Tables S9 and S10).

### 3.8. Analysis of Molecular Docking

**Figure 4:**
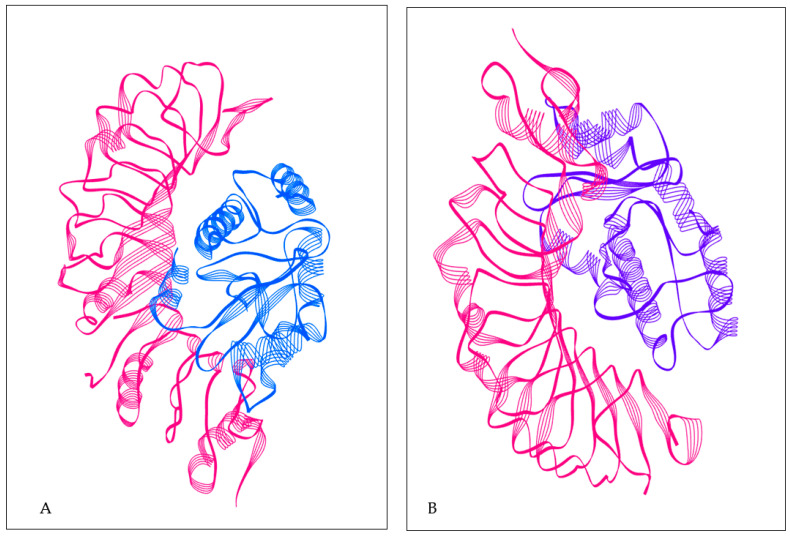
(**A**) TLR2 with constructed vaccine; TLR2 structure in pink color; constructed vaccine in blue color. (**B**) TLR4 with constructed vaccine; TLR4 structure in pink color; constructed vaccine in purple color.

**Figure 5:**
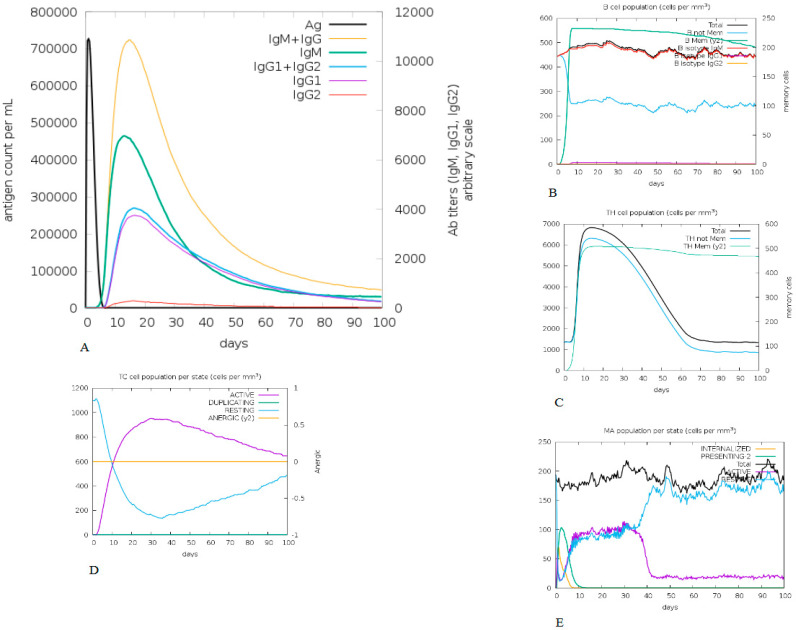
Immune simulation profile of constructed vaccine. (**A**) Exhibits both the relative antibody responses and antigen concentration and the presence of protective IgGs indicates that the vaccination was effective. (**B**) Depicts the relevant number of antibody-producing plasma cells. (**C**–**E**) Presents the activity of macrophages cytotoxic T and helper T cells.

For the stable or long-term immunity of the designed vaccine, it is important that the constructed vaccine has ability to interact molecularly with immune cells. It is already known that Toll-like receptors (TLRs) play an important role in innate immunity. Molecular interaction analyses were conducted with TLR2 and TLR4 to confirm the immune ability of the constructed vaccine (Figure 4 TLR2, Figure 5 TLR4). Molecular interaction analysis was performed by the PatchDock server, and refinement of the best complexes was performed by FireDock. The complexes were selected on the basis of best global energy (−43.65), attractive van der Waals (VdW) energy (−32.23), repulsive VdW energy (12.19), hydrogen bond (HB) energy (−4.81), and atomic contact energy (ACE; 4.08) for TLR2. For TLR4, it was selected on the basis of global energy (−52.36), attractive van der Waals (VdW) energy (−38.74), repulsive VdW energy (17.16), hydrogen bond (HB) energy (−1.81), and atomic contact energy (ACE; 3.26) (Supplementary Table S11).

### 3.9. In Silico Immune Simulation

**Figure 6:**
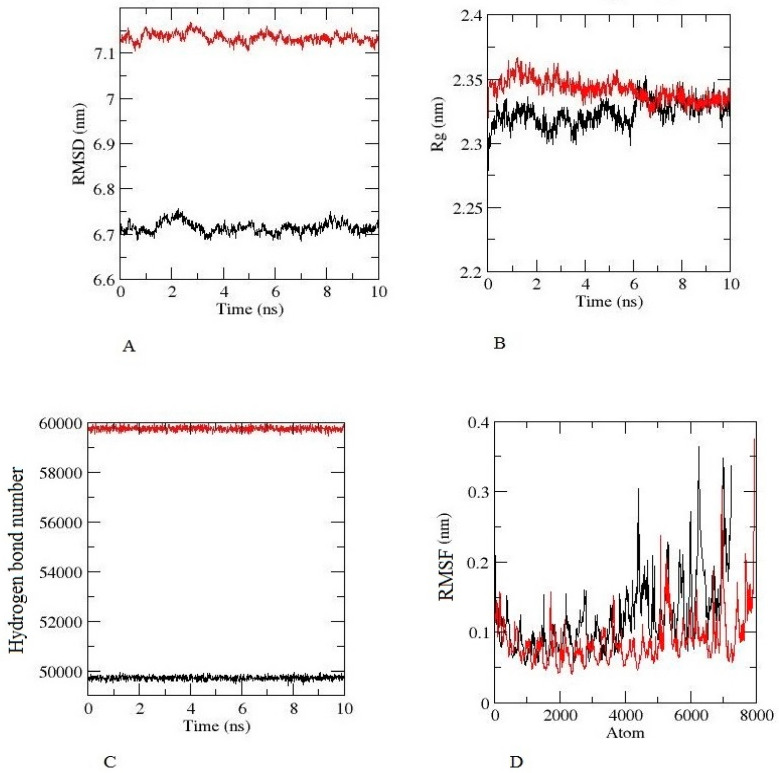
Molecular dynamic simulation results of constructed vaccine, TLR-2 (Black), TLR-2 (Red), for 10 ns (**A**) The RMSD plot (**B**) The Rg (radius of gyration) plot. (**C**) The hydrogen bond plot of constructed vaccine and TLRs docked complexes. (**D**) RMSF profile of docked complexes.

The constructed vaccine immunogenic profile is given in Figure 6. The immune simulation results show that, after the antigenic primary responses, IgM + IgG, IgM, IgG1 + Ig G2, Ig G1, Ig G2 responses seemed to have significant antibody titters in Figure 6A,B. The cell isotype predictions are shown in Figure 6B. In addition, a significant response was also observed in CTL and HTL populations (Figure 6C,D, respectively). However, macrophage activity was also found to be significantly improved (Figure 6E). This entire immunogenic profile showed that a constructed vaccine has the ability to develop immune memory cells.

### 3.10. Analysis of Molecular Dynamics and Simulation

The root-mean-square deviation (RMSD) for the TLR-2 and TLR-4 complexes was calculated for the constructed vaccine complexes. The RMSD run of constructed vaccine + TLR2 and constructed vaccine + TLR4 was well equilibrated and showed a minimum deviation of 6 ns.

The equilibration of RMSD values is depicted in Figure 6A. For the assessment of the overall compactness of the TLR2 and TL4 receptors, Rg values were calculated for each complex, as depicted in Figure 6B, Rg values for TLR2 and TLR4 with a constructed vaccine complex was fluctuated at 2.35 nm and decreased to a minimum value of ~2.35 nm. Hydrogen bond formation was further assessed during the entire simulation period, as seen in Figure 6C. The Figure 6D depicts the RMSF value of the constructed vaccine + TLR2 and the constructed vaccine + TLR4. From the Figure, it can be observed that for both complexes, fluctuation was at its minimum, and the complex demonstrated confined movements during molecular dynamics simulation. The Supplemental Video of TLR2 and TLR4 with a constructed vaccine shows that the vaccine was stable and attached to TLR2 and TLR4 throughout the simulation.

## 4. Discussion

According to the WHO, the fatality ratio of monkeypox is ~3 to 5%. Despite this fact, there is no proper treatment strategy for monkeypox [2]. However, in some cases, the smallpox vaccine may provide protection against monkeypox [2]. A specific vaccine may be required for better protection or the eradication of the monkeypox virus infection. In this study, we developed a peptide-based vaccine by using an immunoinformatic approach, which may give researchers strong ideas for the development of antigenic epitopes to design vaccines. In this study, the vaccine was designed to provide immunity by using several antigenic peptide fragments, despite using a whole genome or large length of protein so as to not cause any allergenic responses in the host. In addition, compared to a conventional and single-epitope vaccine, our designed vaccine may have different benefits, such as: the presence of multiple MHC epitopes and T-cell receptors; overlapped CD4^+^ and CD8^+^ T-cell epitopes; addition of multiple epitopes from monkeypox protein; and an adjuvant for long-term immunity. This method has been used previously, resulting in the expansion of protective efficiency in vivo. Additionally, some vaccines developed by this method have been approved for clinical trials [32,33,34].

The interferon gamma, T-cell lymphocyte, and helper T-cell lymphocyte epitope for monkeypox were identified by using several immune filter tools. Only non-allergic sequences and antigenic sequences that were able to interact with different HLA alleles were conserved from different monkeypox strains, and they were not similar to the human proteome to minimize the risk of autoimmunity [35]. The vaccine was designed to be polyepitope (CTL, HTL, IFN with an adjuvant). In addition, GPGPG and AAY linkers were added for the prevention of junctional epitope formation. However, GPGPG and AAY linkers were used as linkers in a previous study and were found to produce junctional immunogenicity in a constructed vaccine. In addition, Arai et al. (2001) reported that the EAAAK linker may improve the bioactivity and stability of the constructed vaccine [35]. In a similar manner, Bazhan et al. (2019) designed a T-cell-based multi-epitope vaccine to combat the Ebola virus. They developed a potential vaccine that was discovered to be immunogenic when produced in mice using antigenic epitopes, predicted by the Immune Epitope Database (IDEB) [20].

Allergies may be an important concern regarding vaccines. In our constructed vaccine, allergenicity was not detected. However, various physicochemical properties of the vaccine were assessed by the ProtParam ExPASy tool, which indicated that the constructed vaccine would be stable (index value of 35.65); the theoretical PI value was found to be 5.71; and the aliphatic index of the vaccine was estimated at 97.17, which is an indication of a thermostable protein. In addition, the GRAVY value was estimated to be 0.073, which indicates that the vaccine may interact with water. Other researchers designed and experimentally validated their constructed vaccines, which were found to be able to produce cellular and humoral immune responses in mice [36]. However, the index value of our constructed vaccine was better than that designed by Foroutan et al. (2020). As per the Ramachandran plot analysis, the constructed vaccine confirmed that 80.7% residues were found in the favored region, 12.7% residues were found in the allowed region, 3% were found in the additionally allowed region, and 3.6% residues were found in the disallowed region, with an ERRAT value of 81.395, indicating that NMR and X-ray crystallographic techniques have already primarily defined most of the protein structure.

It is already known that TLRs are expressed in monocytes, macrophage cells, and granulocytes [37]. The constructed vaccine’s molecular interactions and binding affinity patterns with TLR-2 and TLR-4 were examined using molecular docking techniques. The best dock complex of TLR2 had global energy (−43.65), attractive van der Waals (VdW) energy (−32.23), repulsive VdW energy (12.19), hydrogen bond (HB) energy (−4.81), and atomic contact energy (ACE; 4.08), while TLR4’s best dock complex had global energy (−52.36), attractive van der Waals (VdW) energy (−38.74), repulsive VdW energy (17.16), hydrogen bond (HB) (−1.81), and atomic contact energy (ACE; 3.26), which denotes that the constructed vaccine has significant binding affinities. Furthermore, molecular dynamics simulations were run with constructed vaccine and TLR2 and TLR4 complexes to assess their stability under atomistic conditions. From molecular dynamics simulation results, it was observed that TLR2 and TLR4 with the constructed vaccine were well equilibrated and showed minimum deviation. The equilibration of RMSD values is depicted in Figure 6A. For the assessment of compactness with the constructed vaccine and TLR2 and TLR4 complexes, we calculated the Rg values. As depicted in Figure 6B, there were very few conformational changes in the Rg value for the TLR2 + constructed vaccine complex, which fluctuated at 2.35 nm and decreased to a value of 2.3 nm only, and the TLR4 + constructed vaccine complex, which fluctuated at 2.3 and decreased to a minimum value. This is indicative of the little conformational changes throughout the simulation. For the stability of the protein structure, the hydrogen bond was assessed, and the constructed vaccine complex showed enough hydrogen bonds, indicating its stability, as seen in Figure 6C. For the assessment of movements during simulation, RMSF values were calculated as presented in Figure 6D, there were restricted movements. These findings show that. under the simulated environment, the vaccine complexes were stable and less mobile. In addition, as per the immune simulation study, our developed vaccine candidate could probably provide a suitable immune response after secondary exposure following the final injection, as shown in Figure 5. The immunoinformatic approaches to the design of a multi-epitope vaccine candidate have been used by several researchers [38,39]; therefore, the development of a vaccine using epitopes appears to be able to activate immune cells in the host, which may subsequently trigger the activation of other immune cells via a convoluted signaling cascade [11,38,39,40].

## 5. Conclusions

Multi-epitope vaccines have already proven to be effective at providing protection and producing immunity in vivo, and some are currently undergoing clinical trials. The current study has been completed by immunoinformatic approaches to recognize potential antigenic epitopes for the construction of a vaccine against monkeypox. The vaccine was developed using three types of antigen epitopes from monkeypox: CTL, HTL, and IFN-γ. However, the physiological profile of the constructed vaccine was assessed by computational approaches and the stability of the constructed vaccine was assessed by molecular dynamics. In addition, insilico immune simulation confirmed that the constructed vaccine has the ability to trigger an immune response. Even so, the precise efficiency can only be determined through experimental analysis. The experimental assay might start with the constructed vaccine production and move on to in vitro and in vivo testing. We also suggest additional research on the synthesis and biological effects of the planned multi-epitope vaccination.

## Data Availability

The authors confirm that the data supporting the study’s findings are included in the article.

## Supplementary Materials

The following supporting information can be downloaded at: https://www.mdpi.com/article/10.3390/vaccines10091564/s1, Table S1: Physical and chemical characteristics of the targeted monkey pox sequence; Table S2: Output of Predicted CTL epitopes as per NetCTL 1.2 server; Table S3: A cytotoxic T-lymphocyte epitope that has been chosen. The table’s epitopes are non-toxic, non-allergic, and completely conserved across the target protein sequence (as predicted using the IDEB conservancy tool). Epitopes with IC50 values under 250 were thought to bind appropriately with the corresponding human leukocyte antigen (HLA) alleles. The antigenicity of the epitopes was predicted using the VaxiJen v2.0 server at 0.4 thresholds; Table S4: Details on a specific helper T-lymphocyte epitope used in the vaccine’s development. The epitopes are categorized as strong and weak binders with respective HLA_DRB1 alleles based on the binding score of less than 2% and 10%, respectively; Table S5: Selected interferon-gamma epitopes included in the vaccine constructs. The epitope was predicted by using the IFN-epitope server; Table S6: Refined the best vaccine structures by using Galaxy refine web server; Table S7: Ramachandran plot statistics of the vaccine structure; Table S8: Physico-chemical properties of the final multi-epitope vaccine construct; Table S9: The Linear B-cell epitopes are described in the developed vaccine; Table S10: The developed vaccine had 102 residues that the Ellipro server indicated would fall under the category of discontinuous B-cell epitopes; Table S11: Refinement of the best-docked complexes (TLR4-vaccine, TLR 2-Vaccine) on FireDock; Figure S1: SOPMA result; Figure S2: Model 1; Figure S3: The ERRAT plot of the finalized multi-epitopic vaccine structure; Figure S4: Allergenicity assessment of the final multi-epitope vaccine. The vaccine was found to be non-allergen by (A) AllerTOP v2.0 and (B) Allergen FP v1.0 sever; Figure S5: Signal peptide prediction in the vaccine constructs; Supplementary Video, TLR2 and TLR4 with a constructed vaccine.
