# Supplementary material for: Multi-Epitope-Based Vaccine Candidate for Monkeypox: An In Silico Approach
Source: Vaccines (Basel). 2022 Sep 19;10(9):1564. doi: 10.3390/vaccines10091564 (PMC9504424; doi:10.3390/vaccines10091564)
Supplement: Supplementary file 1 [file vaccines-10-01564-s001.zip › vaccines-1888343-supplementary.pdf]

SUPPLEMENTARY TABLES

Supplementary Table S1. Physical and chemical characteristics of the targeted monkey pox sequence.

Parameter	Value
Molecular weight	35278.02
Extinction coefficients Abs. 0.1% (=1 g/l) 1.595, assuming all pairs of Cys residues form cystines	56270
Ext. coefficient Abs. 0.1% (=1 g/l) 1.595, assuming all Cys residues are reduced	56270
Theoretical pI	7.77
Total number of negatively charged residues (Asp + Glu)	31
Total number of positively charged residues (Arg + Lys)	32
Half-Life	30 hours (mammalian reticulocytes, in vitro).<br>>20 hours (yeast, in vivo)<br>>10 hours (Escherichia coli, in vivo).
Instability index	44.95
Grand average of hydropathicity (GRAVY)	-0.367
Aliphatic index	87.89

Supplementary Table S2: Output of Predicted CTL epitopes as per NetCTL 1.2 server.

Super-types	Epitopes	Position	Prediction score	Allergenicity	Antigenicity score
A1	ITENYRNPY	215	3.09	Non allergen	0.8011
	TTSPVRENY	242	2.71	Non allergen	0.7917
	VSDHKNVYF	123	1.88	Non allergen	1.0903
	GSNHLIDVY	77	1.86	Allergen	-0.0002
	QVSDHKNVY	122	1.53	Allergen	0.2986
	PSTLDYFTY	161	1.5	Non allergen	0.2981
	LNDDTQVYY	225	1.42	Non allergen	-0.126
	YVLSTIHIY	61	1.4	Non allergen	0.5976
	MSAPFDSVF	146	1.33	Non allergen	-0.0184
	NLLPSTLDY	158	1.29	Allergen	1.0771
	SADAAWIIF	177	1.19	Non allergen	0.8244
	HSADAAWII	176	1.18	Non allergen	0.8212
	KLNDDTQVY	224	1	Allergen	-0.3658
	RLKTLDIHY	20	0.92	Non allergen	1.9035
	SAPFDSVFY	147	0.92	Allergen	0.0542
	LSDLREACF	255	0.91	Non allergen	1.7255
	LREACFSYY	258	0.83	Non allergen	1.5067
	YSGEINLVH	87	0.82	Allergen	0.8764
	ILFLMSQRY	290	0.79	Non allergen	1.0469
A2	FILTAILFL	285			
	STLDYFTYL	162	0.12	Non allergen	0.1219
	ILTAILFLM	286	0.14	Non allergen	0.1416
	KTFAIIAIV	274	1.03	Non allergen	1.0273
	IIIAIFLQV	115	0.28	Non allergen	0.2816
	YLDNLLPST	155	0.21	Non allergen	0.2106
	FVFILTAIL	283	0.46	Non allergen	0.4639
	FAIIAIVFV	276	1.34	Non allergen	1.3444
	IIFPTPINI	183	0.52	Allergen	0.5175
	NMSAPFDSV	145	0.24	Allergen	0.2394
	GIIIIAIFL	113	0.26	Allergen	0.2618
	QLSPINIET	4	1.74	Allergen	1.7364
	QLSKFRTLL	195	-0.88	Non allergen	-0.8785
A3	ILFLMSQRY	290	1.05	Non allergen	1.0469
	KLNDDTQVY	224	-0.37	Non allergen	-0.3658
	NIHSDQLSK	190	-0.52	Allergen	-0.5227
	RLKTLDIHY	20	1.9	Allergen	1.9035
	AILFLMSQR	289	1.06	Non allergen	1.0611
	FLMSQRYSR	292	0.98	Non allergen	0.9843
	KAISDTRLK	14	1	Non allergen	1.0032
	NLLPSTLDY	158	1.08	Allergen	1.0771
	MSQRYSREK	294	0.02	Allergen	0.0244
	STIHIYWGK	64	1.26	Non allergen	1.2641

	NLVHWNKKK	92	1.23	Non allergen	1.232
	RTLLSSSNH	200	0.01	Allergen	0.0113
	EINLVHWNK	90	1.37	Non allergen	1.3749
A24	VFILTAILF	284	1.8027	Non allergen	0.3203
	EYVLSTIHI	60	1.6367	Non allergen	0.74
	YFQKIVNQL	130	1.4702	Allergen	-0.5633
	TFAIIAIVF	275	1.4664	Non allergen	1.1105
	KYIEGNKTF	268	1.386	Allergen	-0.5044
	AWIIFPTPI	181	1.3567	Allergen	0.1356
	YFTYLGTTI	166	1.334	Non allergen	1.2121
	VFVFILTAI	282	1.1803	Non allergen	0.2176
	KYSGEINLV	86	1.1664	Allergen	0.5484
	AIIAIVFVF	277	1.0901	Non allergen	0.76
	YFMKWLSDL	250	1.0015	Non allergen	-0.835
	GFLPNEYVL	55	0.8704	Allergen	0.4979
	RSANMSAPF	142	0.8239	Non allergen	0.9595
A26	APFDSVFYL	148	1.0945	Allergen	0.2814
	SPVRENYFM	244	1.0464	Non allergen	0.7182
	LPNEYVLST	57	0.7742	Allergen	0.1009
B7	APFDSVFYL	148	1.0945	Allergen	0.2814
	SPVRENYFM	244	1.0464	Non allergen	0.7182
	LPNEYVLST	57	0.7742		
B8	YFQKIVNQL	130	1.3479	Allergen	-0.5633
	QLSKFRTLL	195	1.1875	Allergen	-0.8785
	FVFILTAIL	283	1.0192	Non allergen	0.4639
	WNKKKYSSY	96	0.9608	Non allergen	0.7394
	IAIVFVFIL	279	0.8848	Non allergen	0.9039
	YFMKWLSDL	250	0.8357	Non allergen	-0.835
	TGKLVRINF	39	1.3479	Non allergen	1.0309
B27	VRINFKGGY	43	1.4326	Allergen	2.2055
	LREACFSYY	258	0.9968	Non allergen	1.5067
	RSANMSAPF	142	0.9404	Non allergen	0.9595
	IRAATTSPV	238	0.8217	Allergen	0.4958
B39	YKYSGEINL	85	1.6933	Allergen	0.4526
	KHDDGIIII	109	1.5123	Allergen	0.4287
	ENYRNPYKL	217	1.3055	Non allergen	0.2126
	YFMKWLSDL	250	1.238	Non allergen	-0.835
	YFQKIVNQL	130	1.2342	Allergen	-0.5633
	FVFILTAIL	283	1.0181	Allergen	0.4639
	NHEGKPHYI	207	0.9476	Allergen	0.645

	IAIVFVFIL	279	0.7937	Non allergen	0.9039
	IHSDQLSKF	191	0.7821	Allergen	-0.6628
B44	RNEYFMKWL	247	1.7064	Non allergen	0.1141
B58	MSAPFDSVF	146	1.9014	Non allergen	-0.0184
	RSANMSAPF	142	1.7392	Non allergen	0.959
	VLSTIHIYW	62	1.7351	Non allergen	1.2426
	TTSPVRENY	242	1.4866	Non allergen	0.7917
	IAIVFVFIL	279	1.447	Non allergen	0.9039
	AIIAIVFVF	277	1.1929	Non allergen	0.76
	SADAAWIIF	177	1.027	Non allergen	0.8244
	HSADAAWII	176	0.9919	Non allergen	0.8212
	VSDHKNVYF	123	0.8657	Non allergen	1.0903
	GSNHLIDVY	77	0.8267	Non allergen	-0.0002
	KLNDDTQVY	224	0.7861	Non allergen	-0.3658
	INHSADAAW	174	0.7821	Allergen	0.7485
B62	KLNDDTQVY	224	1.438	Non allergen	-0.3658
	YVLSTIHIY	61	1.3805	Non allergen	0.5976
	RSANMSAPF	142	1.3512	Non allergen	0.9595
	MSAPFDSVF	146	1.34	Non allergen	-0.0184
	RLKTLDIHY	20	1.3211	Non allergen	1.9035
	ILFLMSQRY	290	1.3062	Allergen	1.0469
	QVSDHKNVY	122	1.2635	Non allergen	0.2986
	AIIAIVFVF	277	1.2462	Non allergen	0.76
	WNKKKYSSY	96	1.1426	Non allergen	0.7394
	ITENYRNPY	215	1.1355	Non allergen	0.8011
	DLREACFSY	257	1.0703	Allergen	2.22258
	NLLPSTLDY	158	1.0664	Allergen	1.077
	GSNHLIDVY	77	1.0186	Allergen	-0.0002
	KYIEGNKTF	268	0.9892	Allergen	-0.5044
	LVHWNKKKY	93	0.9244	Non allergen	1.041
	VFILTAILF	284	0.8808	Allergen	0.3203
	SNHEGKPHY	206	0.8546	Non allergen	0.717
	FVFILTAIL	283	0.846	Non allergen	0.4639
	TTSPVRENY	242	0.8293	Non allergen	0.7917
	TFAIIAIVF	275	0.8047	Allergen	1.1105

Supplementary Table S3. A cytotoxic T-lymphocyte epitope that has been chosen. The table's epitopes are non-toxic, non-allergic, and completely conserved across the target protein sequence (as predicted using the IDEB conservancy tool). Epitopes with IC50 values under 250 were thought to bind appropriately with the corresponding human leukocyte antigen (HLA) alleles. The antigenicity of the epitopes was predicted using the VaxiJen v2.0 server at 0.4 thresholds.

Epitopes	Position	HLA class 1 super types (Combined score)	MHC Class 1 alleles	IC50	Antigenicity
VSDHKNVYF	123	A1(1.8)	HLA-C*05:01	3.4	1.09
VSDHKNVYF	123		HLA-C*01:01	242	1.09
VSDHKNVYF	123		HLA-C*07:01	139	1.09
VSDHKNVYF	123		HLA-C*12:03	23.50	1.09
VSDHKNVYF	123		HLA-C*01:01	242	1.09
RLKTLDIHY	20	A1(0.92)	HLA-B*12:03	8.7	0.92
RLKTLDIHY	20		HLA-B*15:01	82.98	0.92
RLKTLDIHY	20		HLA-B*30:02	288.7	0.92
RLKTLDIHY	20		HLA-B*14:02	301.03	0.92
RLKTLDIHY	20		HLA-B*15:01	82.98	0.92
LSDLREACF	255	A1(0.90)	HLA-C*05:01	1.255	1.25
LSDLREACF	255		HLA-C*12:03	17.50	1.25
LSDLREACF	255		HLA-C*14:02	284.85	1.25
LSDLREACF	255		HLA-C*08:02	421.85	1.25
LSDLREACF	255		HLA-B*15:02	345.32	1.25
LREACFSYY	258	A1(0.80)	HLA-C*12:03	258	1.50
LREACFSYY	258		HLA-C*14:02	258	1.50
LREACFSYY	258		HLA-C*07:01	258	1.50
LREACFSYY	258		HLA-C*06:02	258	1.50
LREACFSYY	258		HLA-C*07:02	258	1.50
ILFLMSQRY	290	A1(0.70)	HLA-C*12:03	20.19	1.04
ILFLMSQRY	290		HLA-A*29:02	54.92	1.04
ILFLMSQRY	290		HLA-B*15:01	92.46	1.04
ILFLMSQRY	290		HLA-C*14:02	132.92	1.04
ILFLMSQRY	290		HLA-B*15:02	197.34	1.04
KTFAIIAIV	274	A2(0.70)	HLA-C*12:03	9.5	1.19
KTFAIIAIV	274		HLA-A*02:06	45.03	1.19
KTFAIIAIV	274		HLA-C*30:01	56.23	1.19
KTFAIIAIV	274		HLA-C*02:01	103.06	1.19
KTFAIIAIV	274		HLA-C*14:02	136.02	1.19
FAIIAIVFV	276	A2(0.35)	HLA-C*12:03	6.8	1.34
FAIIAIVFV	276		HLA-C*02:06	9.17	1.34
FAIIAIVFV	276		HLA-C*03:03	18.13	1.34
FAIIAIVFV	276		HLA-C*02:01	59.44	1.34
FAIIAIVFV	276		HLA-C*15:02	190.29	1.34
ILFLMSQRY	290	A3(1.2)	HLA-A*12:03	20.19	1.04
ILFLMSQRY	290		HLA-A*29:02	54.92	1.04
ILFLMSQRY	290		HLA-B*15:01	92.46	1.04
ILFLMSQRY	290		HLA-C*14:02	132.92	1.04
ILFLMSQRY	290		HLA-B*15:02	197.34	1.04

KAISDTRLK	14	A3(1.05)	HLA-C*12:03	15.71	1.003
KAISDTRLK	14		HLA-A*03:03	38.51	1.003
KAISDTRLK	14		HLA-A*30:01	73.95	1.003
KAISDTRLK	14		HLA-A*11:01	138.13	1.003
KAISDTRLK	14		HLA-A*15:02	349.47	1.003
STIHIYWGK	64	A3(0.9)	HLA-A*68:01	13.41	1.26
STIHIYWGK	64		HLA-A*11:01	13.94	1.26
STIHIYWGK	64		HLA-A*12:03	33.51	1.26
STIHIYWGK	64		HLA-A*03:03	66.16	1.26
STIHIYWGK	64		HLA-A*30:01	125.88	1.26
NLVHWNKKK	92	A3(0.91)	HLA-C*12:03	41.32	1.23
NLVHWNKKK	92		HLA-C*14:02	90.28	1.23
NLVHWNKKK	92		HLA-C*03:03	106.07	1.23
NLVHWNKKK	92		HLA-C*14:02	90.28	1.23
NLVHWNKKK	92		HLA-C*03:03	106.07	1.23
EINLVHWNK	90	A3(0.77)	HLA-C*12:03	34.69	1.3
EINLVHWNK	90		HLA-A*68:01	42.21	1.3
EINLVHWNK	90		HLA-A*05:01	51.72	1.3
EINLVHWNK	90		HLA-A*15:02	151.50	1.3
EINLVHWNK	90		HLA-A*11:01	232.97	1.3
YFTYLGTTI	166	A3(0.15)	HLA-C*03:03	13.32	1.2
YFTYLGTTI	166		HLA-C*14:02	32.85	1.2
YFTYLGTTI	166		HLA-C*12:03	54.22	1.2
YFTYLGTTI	166		HLA-B*15:02	210.97	1.2
YFTYLGTTI	166		HLA-C*08:02	259.53	1.2
SPVRENYFM	244	A26(1.09)	HLA-C*03:03	26.27	0.71
SPVRENYFM	244		HLA-C*12:03	33.51	0.71
SPVRENYFM	244		HLA-B*35:01	90.69	0.71
SPVRENYFM	244		HLA-B*15:02	329.79	0.71
SPVRENYFM	244		HLA-B*07:02	522.26	0.71
WNKKKYSSY	96	B8(0.9)	HLA-C*12:03	4.67	0.73
WNKKKYSSY	96		HLA-C*03:03	68.64	0.73
WNKKKYSSY	96		HLA-B*08:01	173	0.73
WNKKKYSSY	96		HLA-C*14:02	255.63	0.73
WNKKKYSSY	96		HLA-B*08:01	173	0.73
IAIVFVFIL	279	B8(0.88)	HLA-C*03:03	10.92	0.90
IAIVFVFIL	279		HLA-C*12:03	24.72	0.90
IAIVFVFIL	279		HLA-B*15:02	130.08	0.90
IAIVFVFIL	279		HLA-B*58:01	165.31	0.90
IAIVFVFIL	279		HLA-A*02:01	352.46	0.90
VLSTIHIYW	62	B58(1.7)	HLA-C*03:03	33.69	1.2
VLSTIHIYW	62		HLA-C*12:03	48.32	1.2
VLSTIHIYW	62		HLA-C*14:02	62.60	1.2
VLSTIHIYW	62		HLA-B*58:01	127.43	1.2
VLSTIHIYW	62		HLA-C*05:01	200.29	1.2
TTSPVRENY	242	B58(1.4)	HLA-C*12:03	29.45	0.79
TTSPVRENY	242		HLA-C*03:03	41.26	0.79
TTSPVRENY	242		HLA-A*68:01	177.21	0.79
TTSPVRENY	242		HLA-A*29:02	178.55	0.79

TTSPVRENY	242		HLA-C*14:02	205.40	0.79
IAIVFVFIL	279	B58(1.4)	HLA-C*03:03	10.92	0.90
IAIVFVFIL	279		HLA-C*12:03	24.72	0.90
IAIVFVFIL	279		HLA-B*15:02	130.08	0.90
IAIVFVFIL	279		HLA-B*58:01	165.31	0.90
IAIVFVFIL	279		HLA-A*02:01	352.46	0.90
VSDHKNVYF	123	B58(0.86)	HLA-C*05:01	3.43	1.09
VSDHKNVYF	123		HLA-C*12:03	23.50	1.09
VSDHKNVYF	123		HLA-C*07:01	139.04	1.09
VSDHKNVYF	123		HLA-A*01:01	242.40	1.09
VSDHKNVYF	123		HLA-C*14:02	286.82	1.09
RLKTLDIHY	20	B62(1.3)	HLA-B*12:03	8.75	1.90
RLKTLDIHY	20		HLA-B*15:01	82.98	1.90
RLKTLDIHY	20		HLA-B*30:02	288.70	1.90
RLKTLDIHY	20		HLA-B*14:02	301.03	1.90
RLKTLDIHY	20		HLA-B*15:02	335.91	1.90
LVHWNKKKY	93	B62(0.92)	HLA-C*12:03	39.01	1.041
LVHWNKKKY	93		HLA-C*14:02	84.84	1.041
LVHWNKKKY	93		HLA-C*03:03	126.36	1.041
LVHWNKKKY	93		HLA-C*29:02	331.71	1.041
LVHWNKKKY	93		HLA-C*15:02	410.42	1.041

Note- All epitope was predicted as non-toxin

Supplementary Table S4. Details on a specific helper T-lymphocyte epitope used in the vaccine's developmentThe epitopes are categorized as strong and weak binders with respective HLA_DRB1 alleles based on the binding score of less than 2% and 10%, respectively.

Epitopes (position)	MHC class 11 alleles	Binding score (%)	Remarks	Antigenicity
TLDYFTYLGTTINHS (4)	DRB1*01:01	1.7	Strong binder	0.7133
	DRB1*04:01	0.6	Strong binder	
	DRB1*07:01	5.0	Weak binder	
LDYFTYLGTTINHSA (164)	DRB1*01:01	1.0	Strong binder	0.6153
	DRB1*04:01	0.25	Strong binder	
	DRB1*07:01	4	Weak binder	
	DRB1*08:01	6.5	Weak binder	
	DRB1*11:01	9.5	Weak binder	
DYFTYLGTTINHSAD (165)	DRB1*01:01	1.6	Strong binder	0.60
	DRB1*04:01	0.4	Strong binder	
	DRB1*07:01	5.5	Weak binder	
	DRB1*08:01	9	Weak binder	
	DRB1*01:01	1.7	Strong binder	
	DRB1*04:01	0.4	Strong binder	

YFTYLGTTINHSADA (166)	DRB1*07:01	0.4	Weak binder	0.822
	DRB1*08:01	8	Weak binder	

Supplementary Table S5. Selected interferon-gamma epitopes included in the vaccine constructs. The epitope was predicted by using the IFN-epitope server.

Epitopes Sequence	Result	Score	Antigenicity
KKHDDGIIIIAIFLQ	Positive	0.240	0.50
GIIIIAIFLQVSDHK	Positive	0.513	0.487
IIIIAIFLQVSDHKN	Positive	0.295	0.54
LDSIRSANMSAPFDS	Positive	0.04	0.50

Supplementary Figure S1. SOPMA result.

```
       10        20        30        40        50        60        70
        |         |         |         |         |         |         |
EAAAKRLKTLDIHYAAYVSDHKNVYFAAYHSADAAWIITTSPVRENYLSDLREACFGPGPTLDYFTYLGT
hhhhhhhhhhhhhhheehhtttceeeeecccttceeeeeccccchhhhhhhhhhtcccccccceeeehhh
TINHSLDYFTYLGTTINHSADYFTYLGTTINHSADYFTYLGTTINHSADAGPGPKKHDDGIIIIAIFLQG
hhccchhhhhhhtccccccthhhhhhhhhcccctthheeeeeeeecccccccccccccttceeeeeehhtt
IIIIAIFLQVSDHKIIIIAIFLQVSDHKNLDSIRSANMSAPFDS
heeeeeeeeccttteeeeeeeeeeccttchhhhhhhcccccccc
```

```
Sequence length :   184
```

```
SOPMA :
   Alpha helix      (Hh) :    60 is  32.61%
   3_10 helix       (Gg) :     0 is   0.00%
   Pi helix         (Ii) :     0 is   0.00%
   Beta bridge      (Bb) :     0 is   0.00%
   Extended strand  (Ee) :    48 is  26.09%
   Beta turn        (Tt) :    18 is   9.78%
   Bend region      (Ss) :     0 is   0.00%
   Random coil      (Cc) :    58 is  31.52%
   Ambiguous states (?)  :     0 is   0.00%
   Other states          :     0 is   0.00%
```

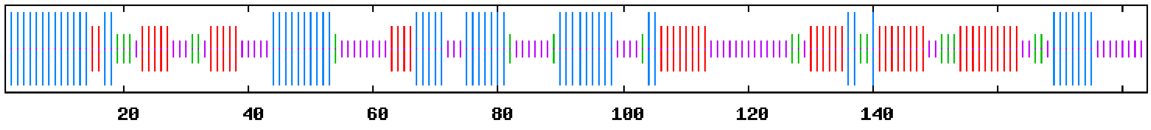

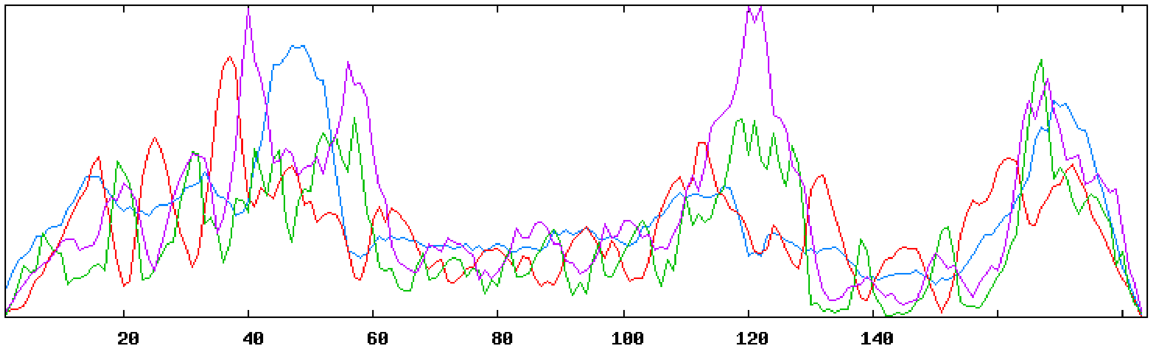


```
Parameters :
   Window width         :  17
   Similarity threshold :   8
   Number of states     :   4
```

Supplementary Figure S2. Model 1.

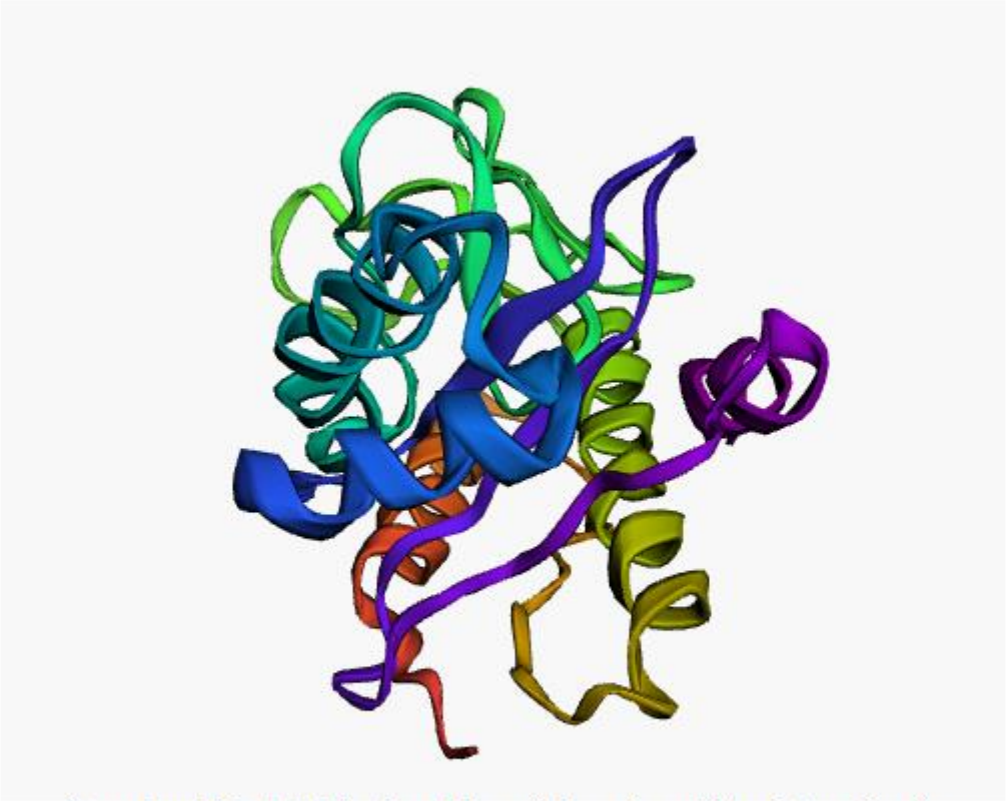

Download Model 1 (colored by rainbow from N to C terminus)
Estimated TM-score: 0.228

Supplementary Table S6: Refined the best vaccine structuresby using Galaxy refine web server.

Model	GDT-HA	RMSD	MolProbity	Clash score	Poor rotamers	Rama favored
Initial	1.0000	0.000	1.974	8.9	1.2	93.2
MODEL 1	0.9688	0.358	2.19	11.8	0.0	87.4
MODEL 2	0.9688	0.350	2.23	12.8	0.6	87.4
MODEL 3	0.949	0.407	2.31	15.3	0.0	86.8
MODEL 4	0.9701	0.356	2.24	13.2	0.0	87.4
MODEL 5	0.968	0.350	2.30	15.3	0.6	87.4

Supplementary Table S7: Ramachandran plot statistics of the vaccine structure.

Plot statistics

Residues in most favoured regions [A, B, L]	134	80.7%
Residues in additional allowed regions [a,b,l,p]	2112.7%	
Residues in generously allowed regions [~a,~b,~l,~p]	5	3.0%
Residues in disallowed regions	6	3.6%
	----	------
Number of non-glycine and non-proline residues	166	100.0%
Number of end-residues (excl. Gly and Pro)	2	
Number of glycine residues (shown as triangles)	10	
Number of proline residues	6	

Total number of residues	184	

Based on an analysis of 118 structures of resolution of at least 2.0 Angstroms and R-factor no greater than 20%, a good quality model would be expected to have over 90% in the most favoured regions.

Supplementary Figure S3: The ERRAT plot of the finalized multi-epitopic vaccine structure.

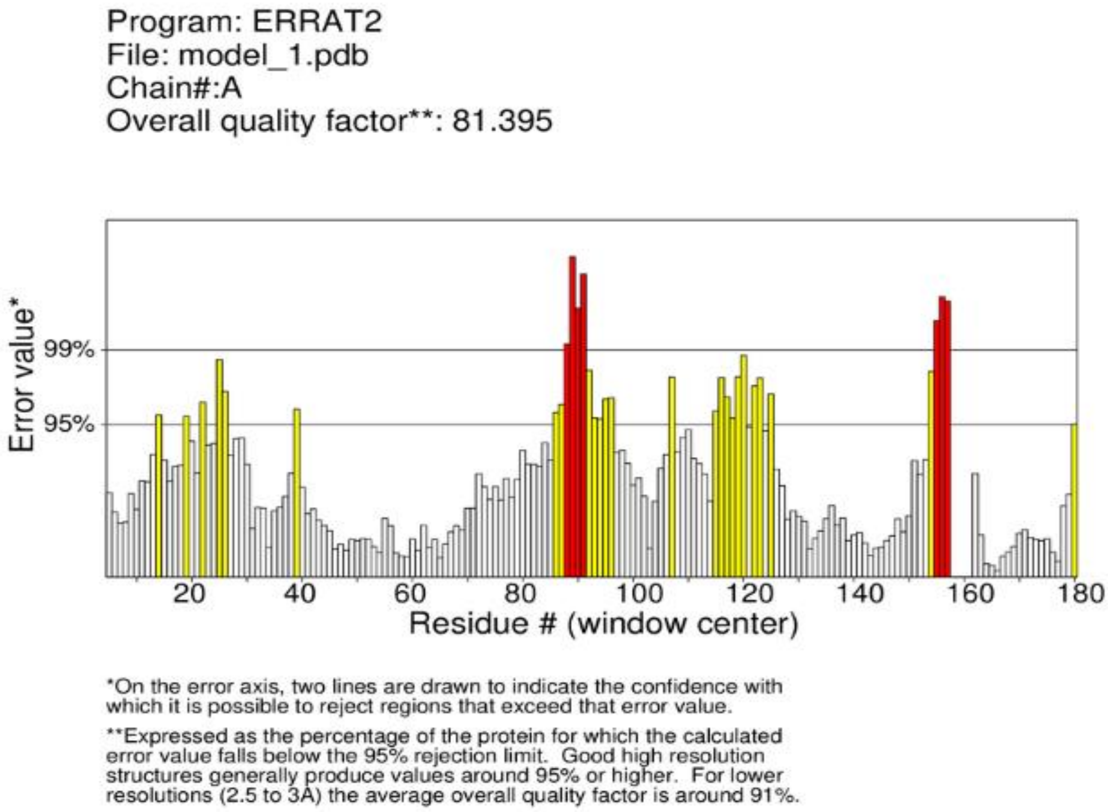

Supplementary Figure S4: Allergenicity assessment of the final multi-epitope vaccine. The vaccine was found to be non-allergen by (**A**) AllerTOP v2.0 and (**B**) Allergen FP v1.0 sever.

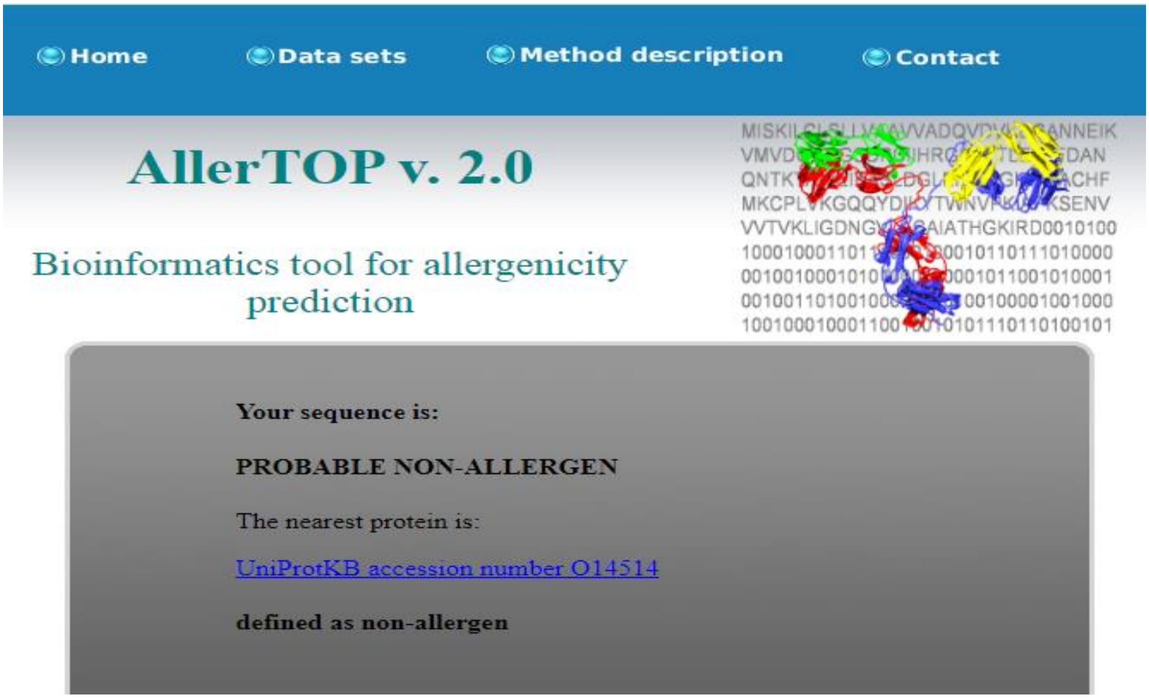

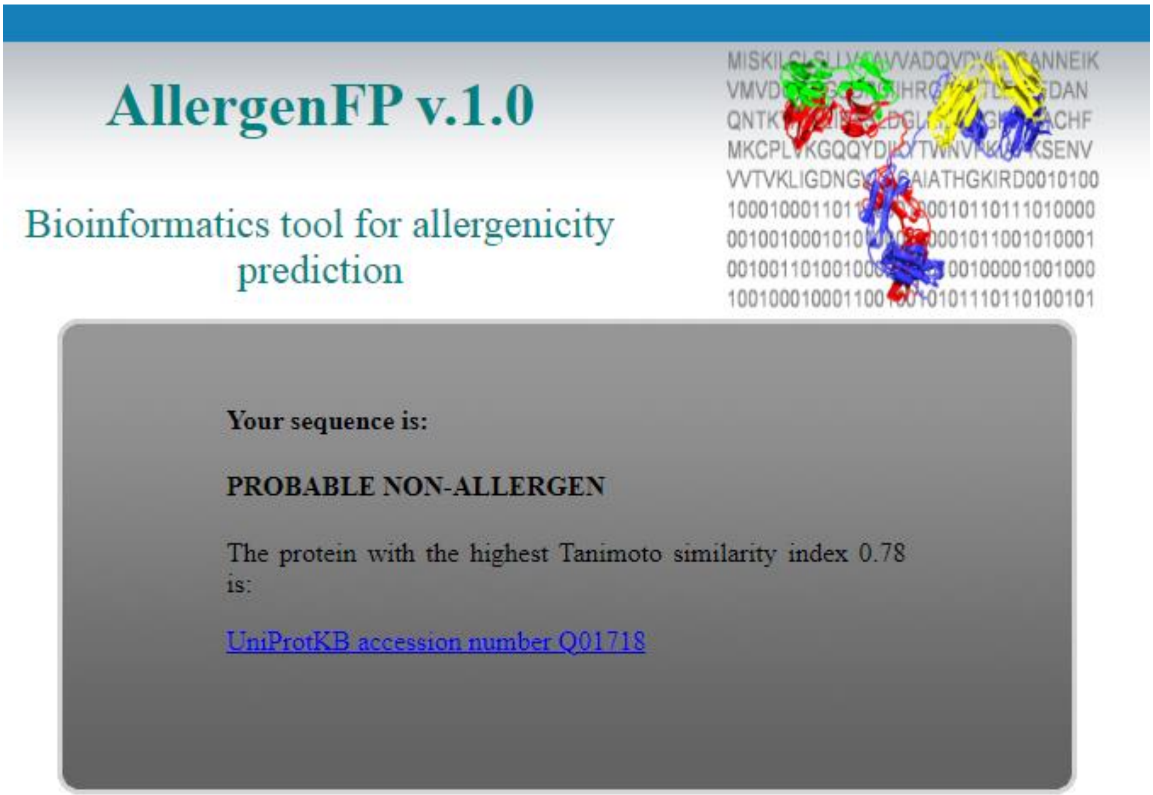

Supplementary Table S8. Physico-chemical properties of the final multi-epitope vaccine construct.

Number of amino acids: 184

Molecular weight: 20430.02

Theoretical pI: 5.71

Amino acid composition:

Ala (A)	20	10.9%
Arg (R)	4	2.2%
Asn (N)	8	4.3%
Asp (D)	15	8.2%
Cys (C)	1	0.5%
Gln (Q)	3	1.6%
Glu (E)	3	1.6%
Gly (G)	10	5.4%
His (H)	10	5.4%
Ile (I)	23	12.5%
Leu (L)	14	7.6%
Lys (K)	7	3.8%
Met (M)	1	0.5%
Phe (F)	10	5.4%
Pro (P)	6	3.3%
Ser (S)	14	7.6%
Thr (T)	16	8.7%
Trp (W)	1	0.5%
Tyr (Y)	13	7.1%
Val (V)	5	2.7%
Pyl (O)	0	0.0%
Sec (U)	0	0.0%

(B)	0	0.0%
(Z)	0	0.0%
(X)	0	0.0%

Total number of negatively charged residues (Asp + Glu): 18
Total number of positively charged residues (Arg + Lys): 11

Atomic composition:

Carbon	C	937
Hydrogen	H	1409
Nitrogen	N	235
Oxygen	O	275
Sulfur	S	2

Formula: $C_{937}H_{1409}N_{235}O_{275}S_{2}$
Total number of atoms: 2858

Extinction coefficients:

Extinction coefficients are in units of M^{-1} cm^{-1}, at 280 nm measured in water.

Ext. coefficient 24870
Abs 0.1% (=1 g/l) 1.217, assuming all pairs of Cys residues form cystines

Ext. coefficient 24870
Abs 0.1% (=1 g/l) 1.217, assuming all Cys residues are reduced

Estimated half-life:

The N-terminal of the sequence considered is E (Glu).

The estimated half-life is: 1 hours (mammalian reticulocytes, in vitro).
30 min (yeast, in vivo).
>10 hours (Escherichia coli, in vivo).

Instability index:

The instability index (II) is computed to be 35.65
This classifies the protein as stable.

Aliphatic index: 97.17

Grand average of hydropathicity (GRAVY): 0.073

Supplementary Figure S5: Signal peptide prediction in the vaccine constructs.

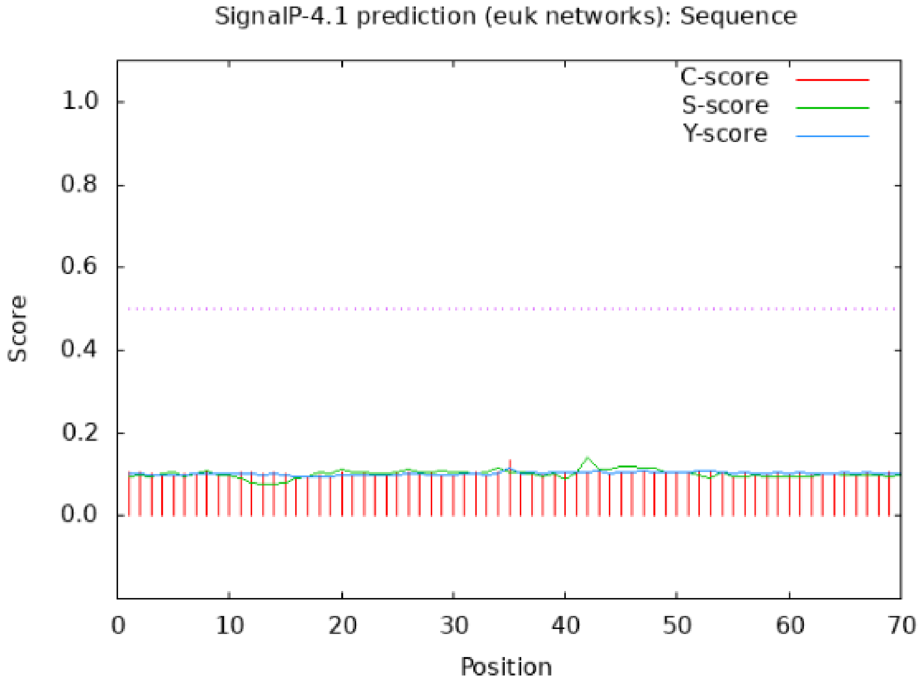


```
# Measure  Position  Value    Cutoff   signal peptide?
  max. C    35       0.135
  max. Y    35       0.117
  max. S    42       0.142
  mean S     1-34    0.100
       D     1-34    0.108   0.450    NO
Name=Sequence   SP='NO' D=0.108 D-cutoff=0.450 Networks=SignalP-noTM
```

Supplementary Table S9: The Linear B-cell epitopes are described in the developed vaccine.

No	Start	End	Peptide	No of residue	Score
1	179	184	SAPFDS	6	0.72
2	8	14	KTLDIHY	7	0.699
3	110	133	LGTTINHSADAGPGPKKHDDGIII	2	0.674
4	43	67	VRENYLSDLREACFGPGPTLDYFTY	25	0.66
5	151	157	SDHKIII	7	0.652
6	102	107	HSADYF	6	0.633
7	19	23	SDHKN	5	0.63
8	160	172	IFLQVSDHKNLDS	13	0.613
9	29	35	YHSADAA	7	0.599
10	88	92	HSADY	5	0.533

Supplementary Table S10: The developed vaccine had 102 residues that the Ellipro server indicated would fall under the category of discontinuous B-cell epitopes.

No	Residues	No of residue	Score
1	A:T9, A:L10, A:D11, A:I12, A:H13, A:Y14, A:V18, A:Y29, A:H30, A:S31, A:A32, A:D33, A:A34, A:A35, A:W36, A:V43, A:E45, A:N46, A:Y47, A:S49, A:D50, A:R52, A:E53, A:A54, A:F56, A:G57, A:P58, A:G59, A:P60, A:T61, A:L62, A:D63, A:Y64, A:F65, A:T66	35	0.665
2	A:S19, A:D20, A:H21, A:K22, A:N23, A:H88, A:S89, A:A90, A:D91, A:Y92, A:N101, A:H102, A:S103, A:A104, A:D105, A:Y106, A:T108, A:L110, A:G111, A:T112, A:T113, A:I114, A:N115, A:H116, A:S117, A:A118, A:D119, A:A120, A:G121, A:P122, A:G123, A:P124, A:K125, A:K126, A:H127, A:D128, A:D129, A:G130, A:I131, A:I133, A:F147, A:L148, A:S151, A:D152, A:H153, A:K154, A:I155, A:I156, A:I157, A:I158, A:Q163, A:V164, A:S165, A:D166, A:H167, A:K168, A:N169, A:D171, A:S172, A:S175, A:A176, A:S179, A:A180, A:P181, A:F182, A:D183, A:S184	67	0.659

Supplementary Table S11: Refinement of the best-docked complexes (TLR4-vaccine, TLR 2-Vaccine) on FireDock.

Rank	Solution Number	Global Energy	Attractive VdW	Repulsive VdW	ACE	HB
1	10	-21.16	-42.23	38.54	9.29	-6.82
2	7	-12.11	-30.60	25.48	7.16	-1.40
3	1	3.67	-0.78	0.02	0.41	0.00
4	4	11.05	-7.50	1.30	3.78	0.00
5	3	29.61	-36.49	21.50	10.85	-2.81
6	2	140.05	-59.29	260.30	11.15	-7.26
7	5	335.96	-53.01	503.52	8.05	-5.90
8	6	558.83	-47.11	774.34	11.72	-5.84
9	9	1589.18	-22.44	1994.18	5.40	-2.08
10	8	1678.01	-44.66	2199.01	2.69	-5.91

VdW (van der Waals), ACE (Atomic Contact Energy), HB (Hydrogen Bonds Energy)
